# Enhanced Poly(propylene carbonate) with Thermoplastic Networks: A Cross-Linking Role of Maleic Anhydride Oligomer in CO_2_/PO Copolymerization

**DOI:** 10.3390/polym11091467

**Published:** 2019-09-08

**Authors:** Lijun Gao, Meiying Huang, Qifeng Wu, Xiaodan Wan, Xiaodi Chen, Xinxin Wei, Wenjing Yang, Rule Deng, Lingyun Wang, Jiuying Feng

**Affiliations:** 1 School of Chemistry and Chemical Engineering, Key Laboratory of Clean Energy Materials Chemistry of Guangdong Higher Education Institutes, Resource and Chemical Engineering Technology Research Center of Western Guangdong Province, Lingnan Normal University, Zhanjiang 524048, China (M.H.) (Q.W.) (X.W.) (X.C.) (X.W.) (W.Y.) (R.D.); 2 School of Chemistry and Chemical Engineering, Key Laboratory of Functional Molecular Engineering of Guangdong Province, South China University of Technology, Guangzhou 510641, China

**Keywords:** poly(propylene carbonate), networks, maleic anhydride oligomer, terpolymerization

## Abstract

Cross-linking is an effective way to enhance biodegradable poly(propylene carbonate) (PPC) from CO_2_ and propylene oxide (PO). Cross-linked PPC can be prepared by one-step terpolymerization of multifunctional third monomers with CO_2_ and PO. However, few such third monomers are available. Each molecule of maleic anhydride oligomer (MAO) contains more than two cyclic anhydride groups. Here, we use it to synthesize PPC with cross-linked networks by adding a small quantity of MAO (0.625–5 wt% of PO) in CO_2_/PO copolymerization that was catalyzed by zinc glutarate. The formation of networks in the prepared copolymers was confirmed by the presence of gel in copolymers combined Fourier transform infrared spectroscopy (FT-IR), ^1^H NMR, and the improved mechanical properties. The 5% weight-loss degradation temperatures and maximum weight-loss degradation temperatures greatly increase up to 289.8 °C and 308.8 °C, respectively, which are remarkably high when compared to those of PPC. The minimum permanent deformation of the copolymers closes to 0, while that of PPC is 173%. The maximum tensile strength of the copolymers is 25.5 MPa higher than that of PPC, reaching 38.4 MPa, and it still has some toughness with the elongation at break of 25%. The above phenomena indicate that MAO that was inserted in PPC chains play a cross-linking role, which results in enhanced thermal stability, dimensional stability, and mechanical strength, comprehensively.

## 1. Introduction

Polypropylene carbonate (PPC) that is synthesized from CO_2_/propylene oxide (PO) copolymerization is a biodegradable polymer material, which has attracted wide attention in the worldwide [1,2]. Over the past few decades, people have focused on the development and commercialization of catalysts. Catalytic efficiency has evolved from the initial turnover frequency (TOF) of less than 1 h^−1^ to tens of thousands h^−1^. Catalytic types have developed from various metal-based to metal-free catalysts [3,4,5,6,7,8,9,10]. For the challenge of PPC large-scale application, besides the need to develop more active catalysts, another important issue is improving the performance of PPC. Its low thermal decomposition temperature cannot make it be heat-processed smoothly. Moreover, the low glass transition temperature (*T*_g_) and amorphism lead to poor mechanical strength and easy deformation. This shortness severely limits its practical application as a viable biodegradable plastic [11]. Therefore, the reinforcement of PPC is urgently needed. So far, many attempts have been made to improve thermal and mechanical properties, such as cross-linking, terpolymerization with co-monomers, and fabrication with other polymers, inorganic fillers, or organic compounds. These methods have been summarized in reviews [11,12,13,14]. In fact, several effective strategies have been implemented. However, the difficulty of PPC modification lies in the comprehensive improvement of PPC properties under the premise of introducing a small amount of other components. Otherwise, the significance of using a CO_2_ resource will be weakened. From this point of view, the crystallization of PPC may be the most ideal fundamental method. Like polyethylene and polypropylene, their *T*_g_s are very low (less than 0 °C), but they can crystallize with a melting point greater than 100 °C. Therefore, they have good dimensional stability and mechanical strength and they can be used as structural materials. However, PPC is very difficult to crystallize. Although various isotactic PPCs have been prepared [15,16], they still cannot crystallize. It is noteworthy that cross-linking has become a very effective modification method for PPC, both physically and chemically. For example, a small quantity (1 wt%) of graphene oxide (Go) nanosheets can greatly enhance PPC. The uniform dispersion and physical cross-linking of Go in PPC matrix are the main mechanisms [17]. Adding 2.5 wt% hyperbranched polyester amide (HBP) in PPC also significantly enhances PPC, in which many hydroxyl/amino groups in HBP formed hydrogen bonds with carbonyl groups in PPC [18]. Here, the physical cross-linking of hydrogen bonds is clear. In addition, cross-linked PPC possesses good thermal stability and mechanical strength, despite having a low *T*_g_. It also has good dimensional stability, especially at elevated temperature, which can effectively solve the cold flow problem of PPC. For instance, 5% weight-loss degradation temperature (*T*_d,−5%_) of 261 °C, maximum weight-loss degradation temperature (*T*_d,max_) of 300 °C, tensile strength of 45.6 MPa, hot-set elongation of 17.3% at 65 °C, and permanent deformation of 0 for chemically cross-linked PPCs have been achieved [19,20]. These properties are more significantly improved than those of PPC.

In the case of PPC cross-linking, there are three typical strategies. The first method is that PPC reacts with various cross-linking agents. For example, triallyl isocyanate (TAIC), diisopropyl peroxide (DCP), [21] and polyvinyl polyphenyl isocyanate (PAPI) [22] were used as cross-linking agents and reacted with PPC to prepare cross-linked PPC. It was also prepared by the addition of polyfunctional monomer, such as TAIC, trimethylopropane triacrylate, pentaerythritol triacrylate, poly(ethylene glycol dimethyl methacrylate), and poly(ethylene glycol dimethyl methacrylate) under electron-beam irradiation [23]. The organosiliconization of PPC using toluene diisocyanate (TDI) and organosilane followed hydrolysis can also get cross-linked PPC [24]. The second method is to introduce double bonds or other functional groups into PPC chains by adding a third monomer in CO_2_/PO copolymerization, followed cross-linking while using initiators, radiation, or cross-linkers. For example, maleic anhydride (MA) [19], allyl glycidyl ether, and vinyl oxide [20,25] were used to copolymerize with CO_2_ and PO, respectively. The prepared copolymers bearing C=C groups were cross-linked by DCP, ultraviolet radiation, and ethylene glycol bis(3-mercaptoproionate) or pentaerythritol tetrakis(mercaptoacetate), respectively. As an alternative, organic silylated PPC was synthesized from CO_2_/ PO/γ-glycidyloxypropyltrimethoxysilane terpolymerization. A cross-linked structure was formed after hydrolysis [26]. Different from the above two-step methods, which are to first prepare PPC followed by cross-linking, the third method is to directly get cross-linked PPC by one-step terpolymerization of CO_2_, PO, and a multifunctional third monomer that usually contains epoxide, anhydride, or isocyanate group, such as vinylcyclohexene dioxide [27], 1,2,7,8-diepoxyoctane, 1,2,9,10-diepoxydecane [28], diphenylmethane diisocyanate [29], pyromellitic dianhydride [30], triglycidyl isocyanurate [31], and bicyclo(2,2,2)oct-7-ene-2,3,5,6-tetracarboxylic dianhydride [32] itaconic anhydride [33]. Our group also prepared PPC with networks in one pot by introducing functional groups, which can react with each other, into the PPC pendants through multi-copolymerization of CO_2_, PO, MA, and furfuryl glycidyl ether [34]. It should be noted that, sometimes, some types of multifunctional third monomers, such as diepoxide [35,36] and polyisocyanate [37], cannot form cross-linking structure by copolymerizing them with PO and CO_2_. This may be related to the reactivity of the monomers and the characteristic of catalysts used.

As mentioned above, we have noticed that, in CO_2_/PO copolymerization, multifunctional epoxide, or isocyanate, third monomers sometimes fail to form cross-linking structure, whereas dianhydrides can do without exception. This is related to the good reactivity of cyclic anhydrides/PO. Various cyclic carboxylic anhydrides have been used as comonomers to polymerize with epoxides in the presence/absence CO_2_ monomer [38,39,40,41,42]. The catalysts used include salen–metal complex, porphyrin complex, double-metal cyanide, (BDI)ZnOAc ((BDI) = β-diiminate), and zinc dicarboxylate. Each of these anhydride monomers only contains one cycoanhydride group, and the polymerization only results in linear polymers. In previous work, we reported PPC with cross-linked networks from CO_2_, PO, and dianhydrides [30,32], which possess good comprehensive properties when compared with PPC. However, within the range of small molecular compounds, multifunctional cyclic anhydride monomers are not as readily available as monofunctional cyclic anhydride monomers, and there have been few reports in this regard [30,32]. Poly(maleic anhydride) (PMA) has multiple cyclic anhydride groups along the backbone. It has been used to build a cross-linking structure in other polymers [43,44]. PMA is commercially available, less expensive, and its hydrolysis products were widely used as dispersants and metal ion binders [45]. Here, we use maleic anhydride oligomer (MAO) as the third multifunctional monomer to copolymerize with PO and CO_2_ for preparing PPC with cross-linked networks in one pot. The thermal stability, dimensional stability, and mechanical properties were fully investigated.

## 2. Materials and Methods

### 2.1. Materials

Toluene was dried over 0.4 nm molecular sieves for more than 24 h before use. Analytically pure benzoyl peroxide (BPO) was recrystallized with chloroform and methanol before use. PO was refluxed over calcium hydride for 8 h, distilled in high pure nitrogen gas flow, and then stored with 0.4 nm molecular sieves. CO_2_ (99.99%) was purchased from Shenzhen Shente Industrial Gas Co. (Shenzhen, China) and directly used. MAO was synthesized according to the literature [46] and modified slightly. Briefly, to a flask were added MA (20 g, 204 mmol) and toluene (23 mL) under a N_2_ atmosphere. When the reaction mixture was stirred and heated to 65 °C, BPO (4 g, 16.5 mmol) dissolved in toluene (11 mL) was added for a period of 1 h. The temperature was then raised to 80 °C and then continued for a further 5 h. The polymer was allowed to separate from the solution and it was washed with hot toluene. Finally, white crystalline MAO was obtained by recrystallization with chloroform. The number molecular weight (*M*_n_) of MAO is 540 g·mol^−1^ with polymer dispersity index of 1.03, which was determined by gel permeation chromatography (GPC) (Figure S1). Zinc glutarate (ZnGA) catalyst was synthesized according to literature [34]. High pure nitrogen gas was purchased from Zhanjiang Oxygen Plant (Zhanjiang, China). All other reagents and solvents are analytical reagents and they were purchased from Shang hai Aladdin Co. (Shanghai, China).

### 2.2. General Copolymerization Procedure

The typical copolymerization was conducted in a 100 mL autoclave that was equipped with a magnetic stirrer. The pre-dried 0.15 g of ZnGA catalyst and a certain proportion of MAO were put into the autoclave and vacuum dried at 100 °C for 8 h. Afterwards, the autoclave was cooled to below 15 °C and carefully cleaned with nitrogen, alternately evacuated three times. Afterwards, 45 mL of PO was injected into the autoclave, and CO_2_ was filled to 2 MPa pressure. When the temperature rose to 70 °C, the pressure of CO_2_ was adjusted to 5.0 MPa. After stirring for 30 h at 70 °C, the autoclave was cooled to room temperature to release pressure. The hard copolymer was dissolved in sufficient acetone containing 5% hydrochloric acid solution, stirred to decompose the catalyst, and then precipitated in distilled water stirred strongly. Such dissolution and precipitation proceeded repeatedly to remove by-product cyclic propylene carbonate (PC) until there was no ^1^H NMR signal of PC, and also to remove hydrochloric acid. The final acetone solution of copolymer was precipitated with ethanol and washed three times in order to reduce the water content of copolymer and facilitate drying. The copolymer was then dried to a constant weight at 80 °C in vacuum, and the yield was calculated.

PPC was also synthesized in the similar procedure to that of copolymers, except that MAO was not added into autoclave.

### 2.3. Characterization and Measurements

^1^H NMR spectra were determined by DRX-400 spectrometer (Bruker Co., Rheinstetten, Germany) with chloroform-*d* as the solvent. Fourier transform infrared spectroscopy (FT-IR) spectra were obtained on a Nicolet 6700 spectrometer (Thermo Fisher Scientific Inc., Waltham, MA, USA) that was equipped with an attenuated total reflection (ATR) accessory. Differential scanning calorimetry (DSC) measurements were conducted in the temperature range of 20–200 °C at a heating rate of 10 °C·min.^−1^ on a Q100 TA instrument (New Castle, DE, USA) under 40 mL·min.^−1^ nitrogen flow. *T*_g_ is defined as the onset of the change of heat capacity. Thermogravimetric analysis (TGA) was measured on a STA 6000 simultaneous thermal analyzer (PerkinElmer Inc., Waltham, MA, USA). The samples were heated from 25–400 °C at a heating rate of 20 °C·min.^−1^ under 40 mL·min.^−1^ nitrogen flow. The average molecular weight of MAO was determined by GPC system (Waters 515 HPLC pump, Waters 2414 detector) with tetrahydrofuran as an eluent. The GPC system was calibrated while using the polystyrene standard with a polydispersity of 1.02.

The gel contents were determined by the ASTM D2765 method. The sample was refluxed in boiled chloroform for 24 h. The insoluble proportion was dried to constant weight at 80 °C in a vacuum. The gel content is defined as the weight percentage of the insoluble proportion in the sample. The data were recorded as the average of three parallel measurements.

The hot-set tests were conducted in an oven. The dumbbell specimens were loaded with 0.14 MPa and the reference length was labeled as *L*_0_ (*L*_0_ = 20 mm). The load specimens were placed in an oven at 60 °C. After 10 min., the length between the marks was measured and recorded as *L*_1_. The load was then released and after 5 min. of relaxation at 60 °C, the specimens were allowed to relax at room temperature until they were no longer shortened. The length between the marks was measured and recorded as *L*_2_. The hot-set elongation and permanent deformation were calculated from (*L*_1_−*L*_0_)/*L*_0_ × 100% and (*L*_2_−*L*_0_)/*L*_0_ × 100%, respectively.

The mechanical properties were tested at 23 °C and 45–55% of humidity with a cross-head speed of 50 mm·min.^−1^ while using an INSTRON 3360 electronic tensile tester (INSTRON Corp. Norwood, MA, USA), according to ASTM D368. The data were recorded as the average value of five parallel determinations. The dumbbell specimens for the tensile tests were prepared by hot embossing, followed by cutting using a dumbbell cutter.

## 3. Results and Discussions

### 3.1. Synthesis

**Scheme 1:**
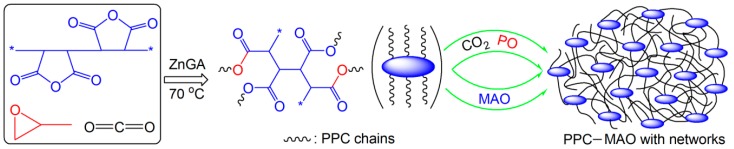
The formation of polypropylene carbonate–maleic anhydride oligomers (PPC–MAOs) with networks in CO_2_/propylene oxide (PO) copolymerization in the presence of MAO.

As mentioned above, cyclic anhydride can copolymerize with epoxides for preparing polyesters while using various types of catalysts. We have prepared cross-linked PPC via one-step CO_2_/PO/dianhydride copolymerization that is catalyzed by ZnGA. Here, MAO is used as a third comonomer in CO_2_/PO copolymerization. MAO feed proportion does not exceed 5 wt% of PO, that is, in the reaction system, CO_2_ and PO are far too much compared to MAO. The *M*_n_ of MAO is 540 g·mol^−1^, which indicates that each molecule contains, on average, five cyclic anhydride groups. When these cyclic anhydride groups ring opening participate in PO/CO_2_ copolymerization, each cyclic anhydride will connect two PPC molecular chains. Therefore, MAO acts as a junction for the formation of cross-linking networks, since it has several cyclic anhydride groups (Scheme 1). The assumption is proved by experiments that the copolymers contain gel. Here we define the copolymers as PPC–MAOx, in which x refers the MAO ratio of PO (wt%). It is obvious that PPC–MAO is composed of linear PPC (CO_2_/PO copolymer) and cross-linked PPC (CO_2_/PO/MAO copolymer). Figure S2 shows the typical photographs of the polymers at the end of the polymerization. When compared with flexible PPC containing a certain quantity of PO, the copolymer looked like a block and felt more elastic when pulled with tweezers. The gel content increased from 14.2–33.6% with increasing MAO feed from 0.625–5 wt% of PO. The copolymer yield increased more than twice (Table 1). In summary of our previous studies on CO_2_/PO/third monomer copolymerization (30–32, 34), it was found that a greater quantity of polymers was obtained when the third monomer was cyclic anhydride, but worse when it was epoxide. This may be related to the higher activity of cyclic anhydride/PO than that of epoxide/CO_2_.

After the copolymerization, the reaction mixture was subjected to a ^1^H NMR test, and mainly contained PPC, PC, and PO based on the ^1^H NMR spectra (Figure S3) [47]. After purification, the ^1^H NMR of the soluble fraction of the copolymer was similar to that of PPC (Figure S4). The PPC selectivity in copolymerization and the carbonate linkages content in PPC chains can be estimated from the integrated area of the signal peaks of PPC and PC. It showed an increase in PPC selectivity and a decrease in carbonate linkages content after adding MAO (Table 1). As the cross-linked networks formed in PPC matrix after introducing MAO, they inhibit the PC formation by the active growth chain terminal alkoxy anion back-biting the carbonyl carbon. Therefore, the PPC selectivity increased with increasing the MAO feed. In addition, No broad signal peaks, such as aromatic end group centered at 7.5 ppm [45] and methine centered at 4.5 ppm [45], were detected in MAO, whether it is the unit inserted into PPC chains or unreacted monomer. Combined with the fact of gel formation, this exhibits that: First, MAO was completely involved in the copolymerization reaction; second, the incorporated MAO units were confined to the gel and they could not be dissolved in the chloroform-*d* solvent. Therefore, we tried to observe the difference between copolymers and PPC by FT-IR spectroscopy. As shown in Figures S5 and S6, the polymer and PPC have approximately the same absorption peaks at 2985 cm^−1^ (asymmetric CH_3_ stretch), 1743 cm^−1^ (asymmetric C=O stretch), 1229 cm^−1^ (asymmetric C(=O)–O stretch), 1165 cm^−1^ (asymmetric C–O–C stretch), 1124 cm^−1^ (symmetric C–O–C stretch), 1067 cm^−1^ (symmetric C(=O)–O stretch), 976 cm^−1^ (CH_3_ out-of-plane bending), and 787 cm^−1^ (CH_2_ out-of-plane bending), which are similar to those of PPC and they are ascribed to carbonate/ester linkages and small amount of ether linkages [30]. After careful identification, it was found that the new characteristic absorption peaks at 1622 cm^−1^ and 701 cm^−1^ appeared in copolymers. They are attributed to the C=C streching vibration and C–H out-of-plane bending vibration of monosubstituted aromatic ring from MAO oligomerized by BPO initiator, respectively. When combined with ^1^H NMR, IR, and the gel formation, it can be inferred that MAO can fully participate in CO_2_/PO copolymerization to form a cross-linked networks.

### 3.2. Thermal Properties

**Figure 1:**
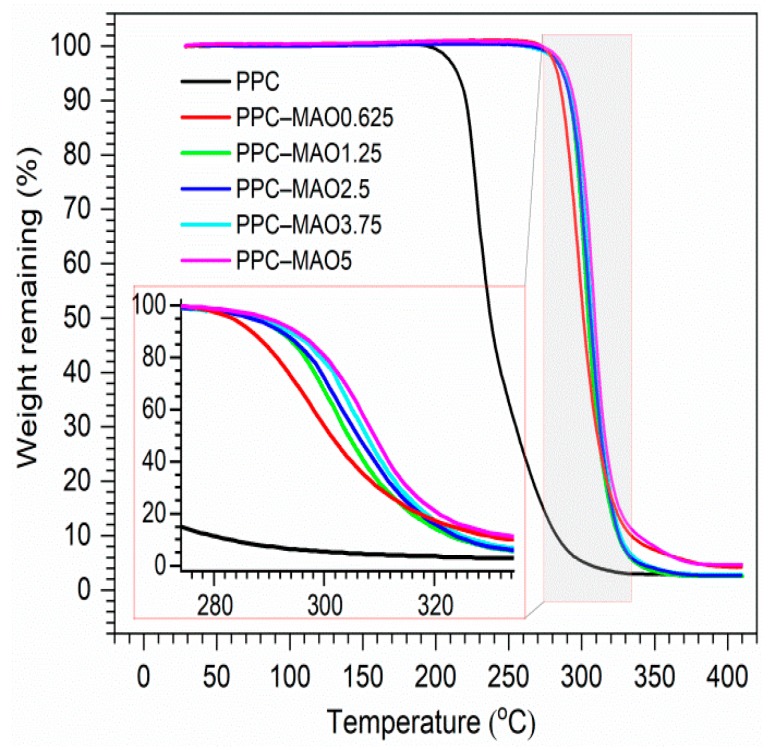
The Thermogravimetric analysis (TGA) curves for PPC and PPC–MAOs with networks.

**Figure 2:**
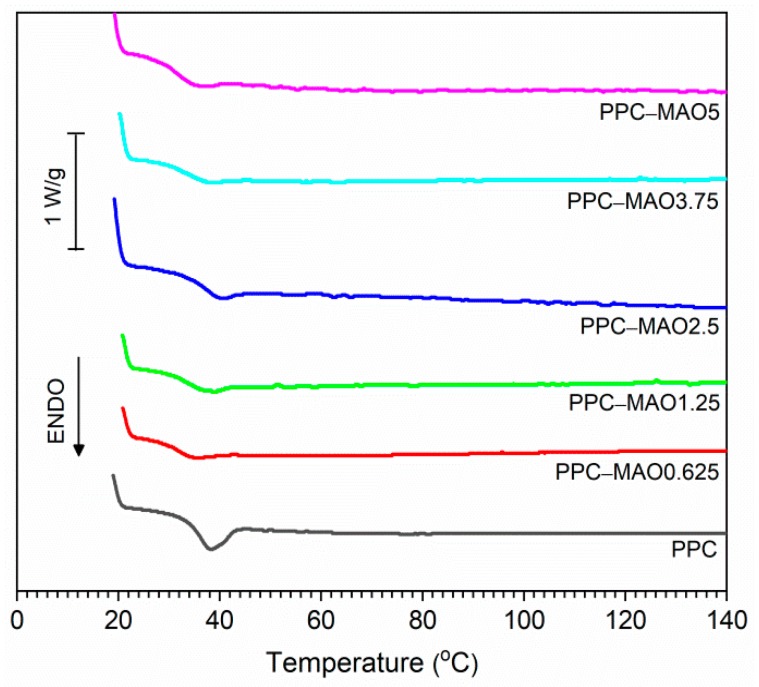
The differential scanning calorimetry (DSC) curves for PPC and PPC–MAO with networks.

Figure 1 and Figure S7 show TGA and DTG curves. The TGA data show that PPC started to lose weight from 195 °C, which is 40 °C higher than 150 °C, as reported in the literature [9], which is related to faster heating rate in TGA test besides polymer molecular structure, like ether linkages content. The copolymers began to lose weight at a temperature above 260 °C, and some such as PPC–MAO3.75 and PPC–MAO5 reached a temperature of 270 °C. For PPC, the 5% weight-loss degradation temperature (*T*_d,__−5%_) was 215.0 °C, and there were two maximum weight-loss degradation temperatures (*T*_d,max_s) of 228.7 °C and 256.0 °C (Table 2). According to the decomposition mechanism of PPC [48], the above two *T*_d,max_s are derived from chain scission and unzipping reaction, respectively. Whereas, the *T*_d,__−5%_ and *T*_d,max_ of each copolymer is over 280 °C and almost 300 °C, respectively (Table 2). This significant improvement in thermal stability is attributed to the formation of cross-links in the PPC matrix by introducing MAO into PPC chains, even with minimal MAO feed (0.625 wt % of PO), since the cross-linking significantly limits the unzipping reaction. Combining our previous studies on the preparation of cross-linked PPC by terpolymerization of multifunctional third monomers with CO_2_ and PO [30,31,32,34], we find that adding a small amount of multifunctional third monomer can significantly increase the thermal decomposition temperature without exception. Suppressing the unzipping degradation plays a key role. As reported by other groups, end-capping by converting PPC’s end-hydroxyl groups into other groups, which reduces the unzipping degradation, can improve the decomposition temperature [49,50]. It is seen that changing the MAO feed has minimal effect on the thermal decomposition temperature under the experimental conditions. The thermal decomposition temperature should gradually increase with less MAO feed. If we only focus on the thermal stability of PPC, it is necessary to find the minimum quantity of MAO. However, the mechanical strength is poor when MAO feed is low, so we did not use a smaller quantity of MAO for the polymerization in order to balance the two properties. In addition, under the experimental conditions, the *T*_g_s first increased and then decreased with increasing MAO feed, but the change is not significant (Figure 2, Table 2). This is related to two contradictory factors. First, introducing flexible MA structural units has a negative impact on *T*_g_ [34,51] and, second, cross-linking has a positive one [19,20,30,34].

### 3.3. Mechanical Properties

**Figure 3:**
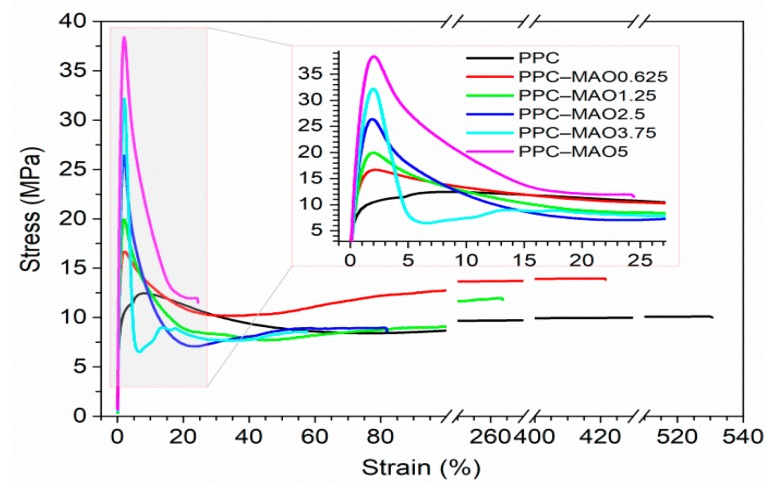
The strain-stress curves for PPC and PPC–MAO with networks.

The tensile tests were conducted to determine the mechanical properties of copolymers. Figure 3 shows the strain-stress curves, and Table S1 lists the data. The tensile strength was up to 38.4 MPa, which is 2× higher that of PPC. Although the elongation at break was reduced, it was still tens of percent, retaining a certain degree of toughness. As the MAO increased, that is, the gel contents increased (Table 1), the tensile strength gradually become higher and, accordingly, the elongation at break decreased. It has been reported that MA was introduced into PPC chains, and the tensile strength of the prepared polymers decreased sharply before cross-linking treatment [51] and increased significantly after cross-linking [19]. In this work, the increase of tensile strength indicates that cross-linked networks really were indeed formed after adding MAO, otherwise the tensile strength would not increase. The increase in gel content suggests that the degree of cross-linking is gradually increasing, and it can be seen that cross-linking can obviously improve the mechanical strength of PPC. The PPC chains mainly contain carbonate linkages and a small quantity of ether linkages. The polar groups are few, the interaction between chains is small, and they are easy to move, so the mechanical strength is weak. When cross-linked networks are contained in PPC matrix, the movement of the chains is restricted, so that the mechanical strength can be improved.

### 3.4. Dimentional Stability

**Figure 4:**
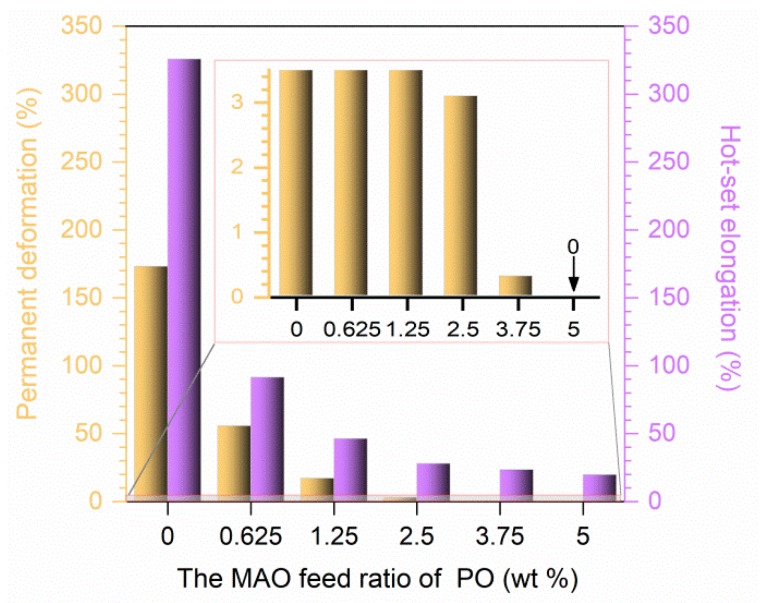
The hot-set elongation and permanent deformation of PPC and PPC–MAO with networks.

The low *T*_g_ and amorphous nature of PPC not only make it weak in mechanical strength, but also easily deform. For example, the poor heat resistance of PPC causes it to soften and deform when held in the hand. However, maintaining the dimensional stability of PPC above 60–70 °C is critical for many applications [35]. The hot-set tests were conducted to determine the dimensional stability. As shown in Figure 4, the hot-set elongation and permanent deformation of PPC–MAOs decreased sharply when compared with PPC. The former decreased from more than 300% to tens of percent, a decrease of 94%; the latter also rapidly decreased from 173% to 55.7%, 17.2%, 3.1%, 0.3%, until 0 with increasing MAO feed from 0–5 wt% of PO. Although the *T*_g_s of PPC–MAOs are lower than that of PPC (Table 1), the hot deformation is much smaller than that of PPC, which fully illustrates the existence of cross-linked networks. Based on the relationship between MAO feed and gel content, the permanent deformation dropped to a few percent when the gel content reached 20%; it approached to 0 when close to 30%. The photographs of dumbbell specimens before and after hot-set test are shown in Figure S8. This significant reduction of permanent deformation indicates that the cross-linked networks were formed after adding MAO, and they enable the copolymers to have stronger resistance to strain and deformation than PPC at a higher temperature.

## 4. Conclusions

PPC with cross-linked networks was synthesized by one step terpolymerization of MAO, PO, and CO_2_ with ZnGA as catalyst. Thus, the prepared copolymers are thermoplastic and have significantly enhanced thermal, mechanical, and dimensional stability. MAO is an inexpensive and readily available multifunctional monomer. Each MAO molecule contains several cyclic anhydride groups which act as cross-linking role in the copolymerization, and the above comprehensive performance enhancements are attributed to the presence of cross-linked networks in the PPC matrix. When combined with our previous work [30,31,32,34], we find that the thermal decomposition temperature can be remarkably increased when a multifunctional third monomer feed proportion is very small. However, relatively more quantity of third monomer is required to significantly improve the mechanical strength. Moreover, the strength and toughness are not only related to cross-linking, but also to the rigidity and flexibility of the third monomer structure. Here, we use the homopolymer of MA. If the copolymer oligomers of MA with various type monomers, such as rigid styrene or flexible methyl vinyl ether, are used as the third multifunctional monomers, it will provide a wide range of ways to balance the mechanical strength and toughness of the cross-linked PPC to meet the needs of different applications.

## Figures and Tables

**Table 1:** The results of copolymerization.

Sample	MAO Ratio of PO (wt%)	Yield (g Polymer/g ZnGA)	Gel (%)	Selectivity ^a^ (% PPC)	Carbonate Linkages ^a^ (%)
PPC	0	26	0	96.4	98.2
PPC–MAO0.625	0.625	53	14.2 ± 1.2	96.7	96.2
PPC–MAO1.25	1.25	58	17.5 ± 1.3	96.6	95.5
PPC–MAO2.5	2.5	64	21.4 ± 1.6	98.3	94.4
PPC–MAO3.75	3.75	67	27.3 ± 2.1	98.3	94.6
PPC–MAO5	5	72	33.6 ± 2.3	97.9	94.1

^a^ Determined by using ^1^H NMR spectroscopy. These data only represent the soluble fraction, excluding the gel, because the gel can not be dissolved in chloroform-*d*.

**Table 2:** The thermal properties of PPC and PPC–MAOs with networks.

Sample	*T*_d,__−__5%_ (°C)	*T*_d,max_ (°C)	*T*_g_ (°C)
PPC	215.0	228.7, 256.0	35.5
PPC–MAO0.625	283.4	297.9	31.8
PPC–MAO1.25	287.1	300.3	34.4
PPC–MAO2.5	287.3	302.5	36.2
PPC–MAO3.75	287.7	302.5	33.7
PPC–MAO5	289.8	308.8	32.2

## Supplementary Materials

The following are available online at https://www.mdpi.com/2073-4360/11/9/1467/s1, Figure S1: GPC curve of MAO, Figure S2: The photographs of (a) PPC and (b) PPC–MAO2.5 at the end of the polymerization, Figure S3: The 1H NMR spectrum of the reaction mixture after CO2/PO/MAO copolymerization (2.5 wt% MAO of PO was used), Figure S4: The 1H NMR spectra of PPC (upper) and PPC–MAO2.5 (below) after purification, Figure S5: The FT-IR spectrum of PPC, Figure S6: The FT-IR spectrum of PPC–MAO2.5, Figure S7: The DTG curves for PPC and PPC–MAOs with networks, Figure S8: The photographs of dumbbell specimens before (upper) and after (below) hot-set test. (a) PPC, (b) PPC–MAO5. The photo below is the permanent deformation, Table S1: The tensile results of PPC and PPC–MAOs with networks.
