# Supplementary material for: Enhanced Poly(propylene carbonate) with Thermoplastic Networks: A Cross-Linking Role of Maleic Anhydride Oligomer in CO2/PO Copolymerization
Source: Polymers (Basel). 2019 Sep 8;11(9):1467. doi: 10.3390/polym11091467 (PMC6780336; doi:10.3390/polym11091467)
Supplement: Supplementary file 1 [file polymers-11-01467-s001.pdf]

Supporting Information for

Enhanced poly(propylene carbonate) with thermoplastic networks: A cross-linking role of maleic anhydride oligomer in CO_2/PO copolymerization

Lijun Gao [1], Meiying Huang [1,2], Qifeng Wu [1], Xiaodan Wan [1], Xiaodi Chen [1], Xinxin Wei [1], Wenjing Yang [1], Rule Deng [1], Lingyun Wang [2] and Jiuying Feng [1,*]

[1] School of Chemistry and Chemical Engineering, Key Laboratory of Clean Energy Materials Chemistry of Guangdong Higher Education Institutes, Resource and Chemical Engineering Technology Research Center of Western Guangdong Province, Lingnan Normal University, Zhanjiang, P. R. China. 524048;

[2] Key Laboratory of Functional Molecular Engineering of Guangdong Province, School of Chemistry and Chemical Engineering, South China University of Technology, Guangzhou , P. R. China. 510641.

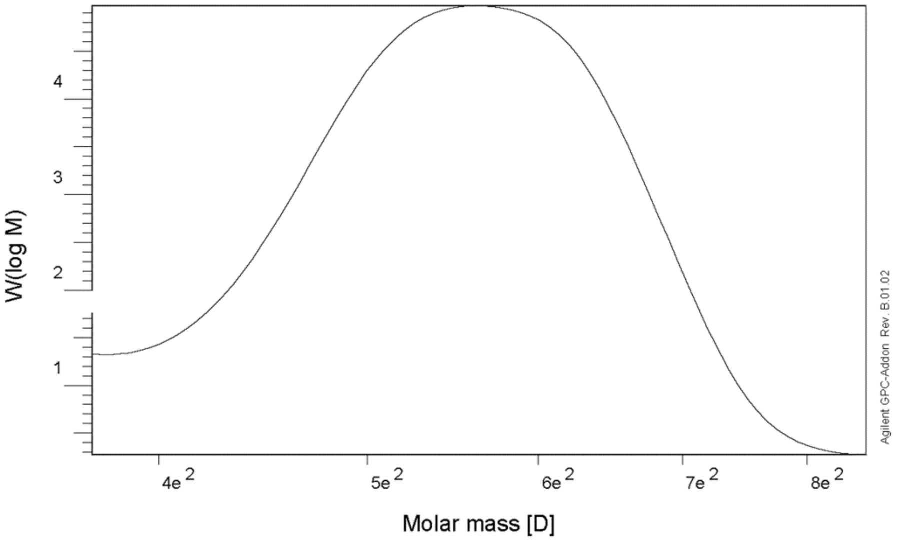

Figure S1. GPC curve of MAO.

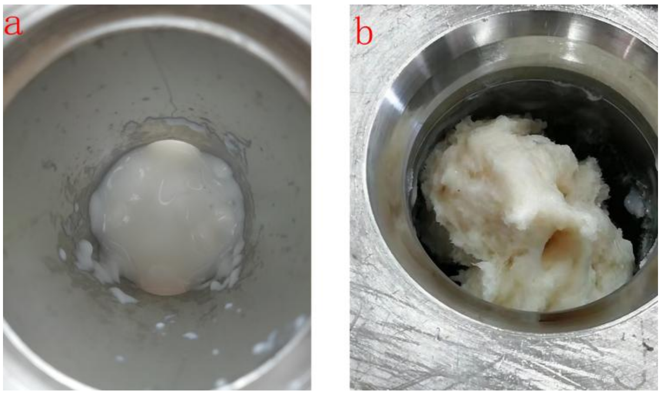

Figure S2. The photographs of (a) PPC and (b) PPC－MAO2.5 at the end of the polymerization.

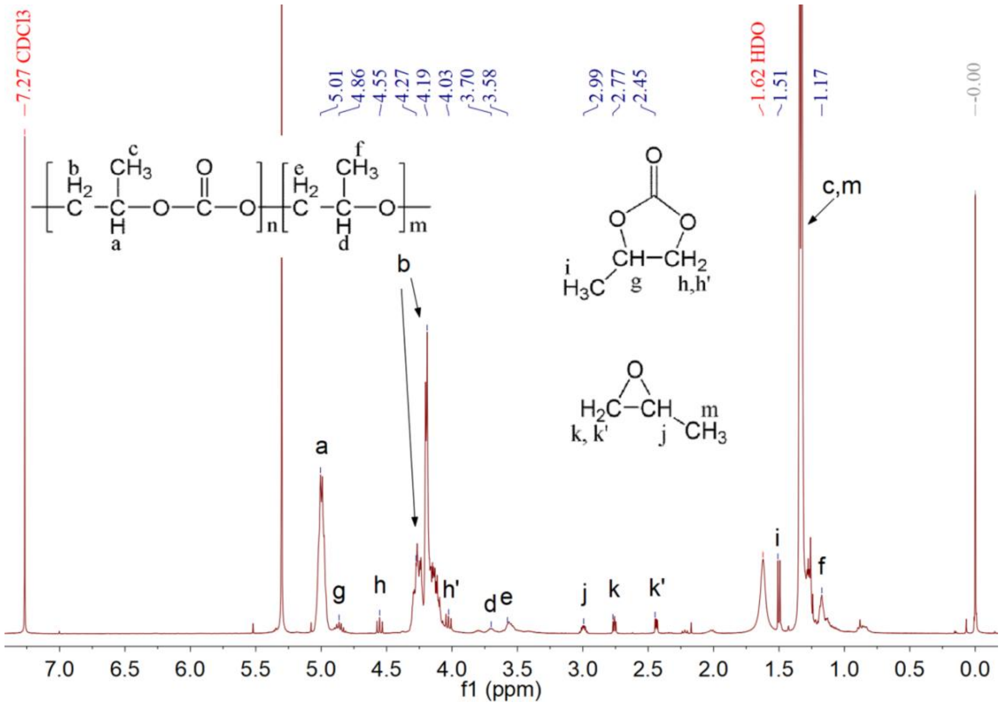

Figure S3. The ^{1}H NMR spectrum of the reaction mixture after CO_2/PO/MAO copolymerization (2.5 wt % MAO of PO was used).

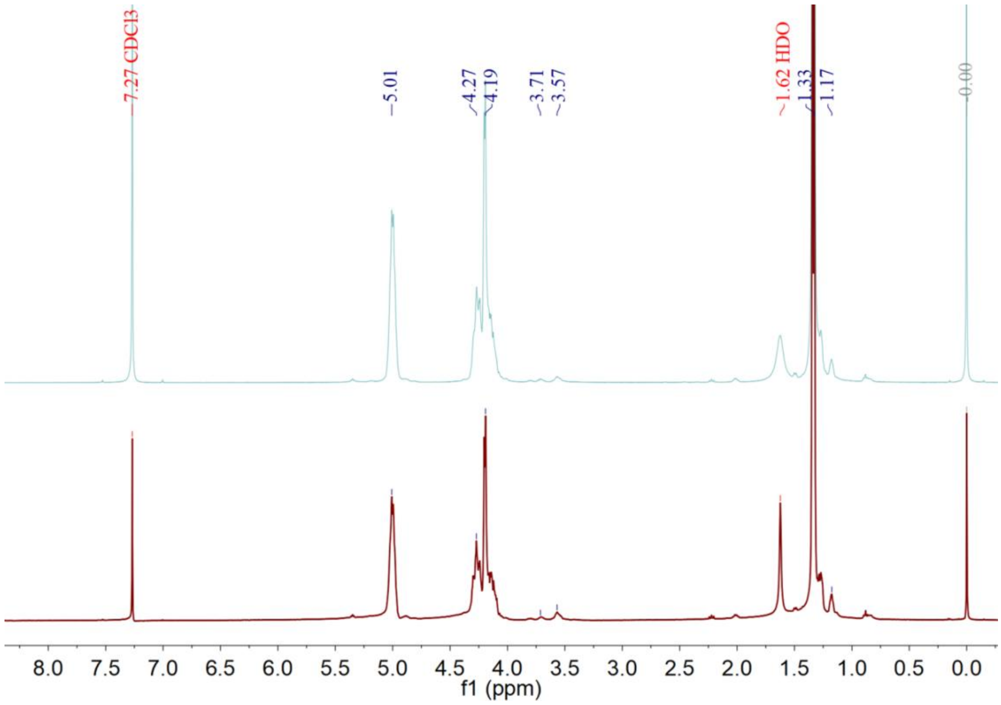

Figure S4. The ^{1}H NMR spectra of PPC (upper) and PPC – MAO2.5 (below) after purification.

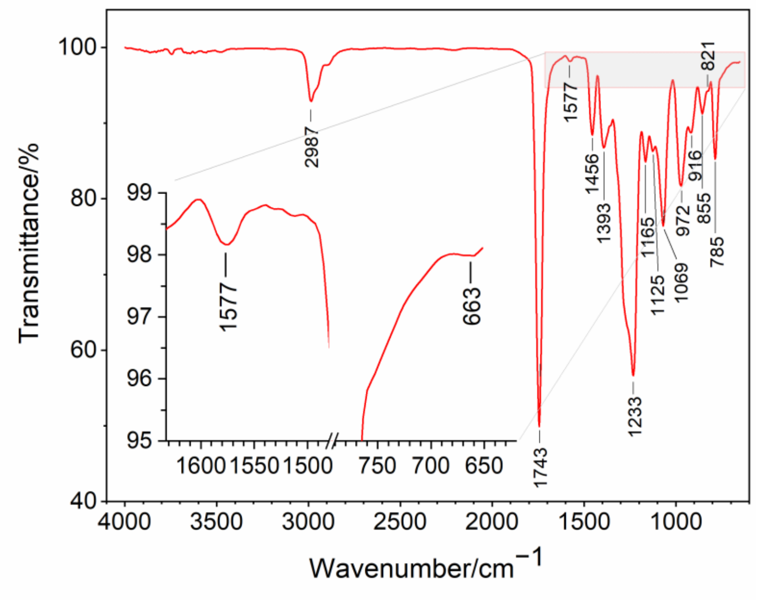

Figure S5. The FT-IR spectrum of PPC.

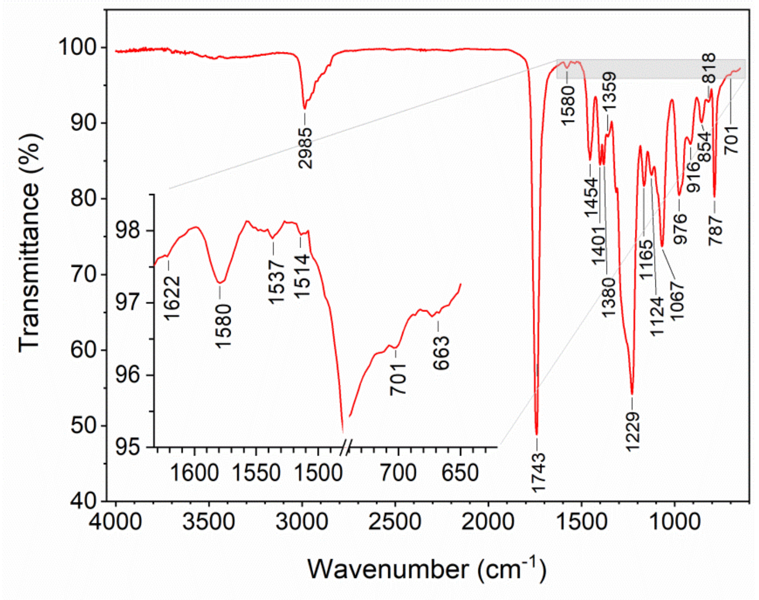

Figure S6. The FT-IR spectrum of PPC－MAO2.5.

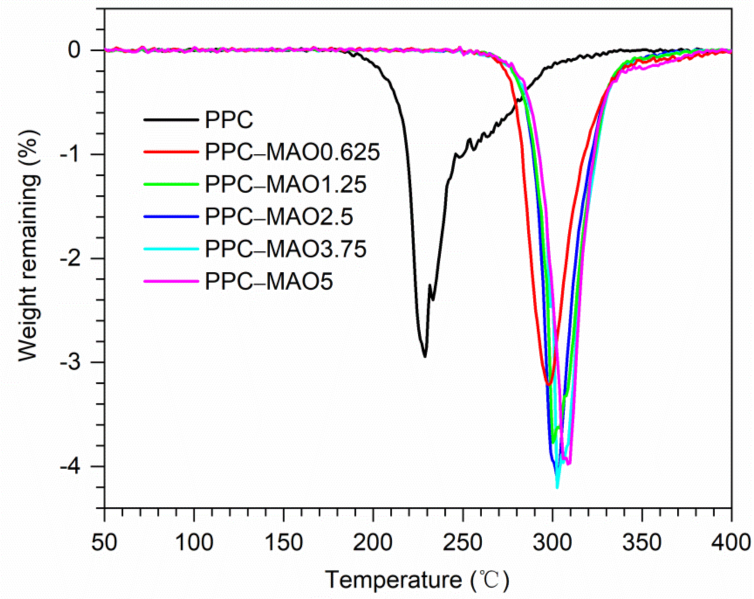

Figure S7. The DTG curves for PPC and PPC – MAOs with networks.

Table S1. The tensile results of PPC and PPC – MAOs with networks.

Sample	Tensile strength/MPa	Elongation at break/%
PPC	12.5±1.4	528±13
PPC – [illegible]	16.8±1.5	422±10
PPC – MAO1.25	20.0±1.9	263±8
PPC – MAO2.5	26.4±2.1	82±4
PPC – MAO3.75	32.1±2.5	58±3
PPC – MAO5	38.4±3.1	25±2

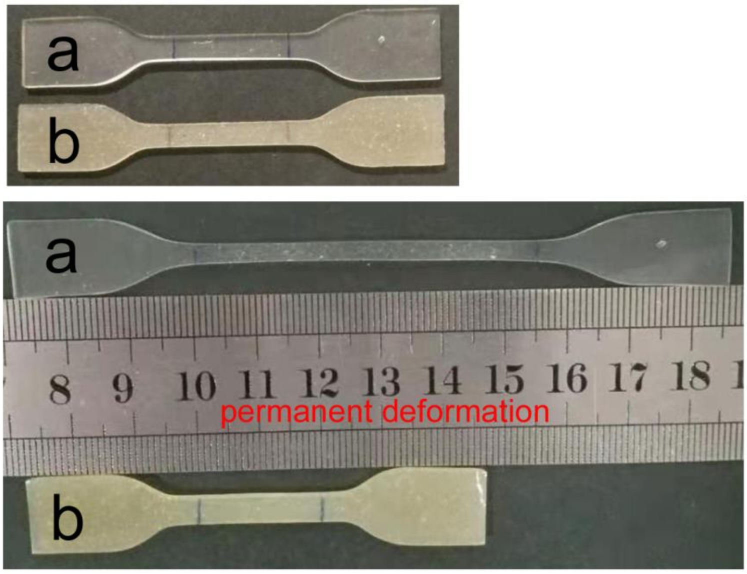

Figure S8. The photographs of dumbbell specimens before (upper) and after (below) hot-set test. (a) PPC, (b) PPC – MAO5. The photo below is the permanent deformation.
